# Early Mortality and AIDS Progression Despite High Initial Antiretroviral Therapy Adherence and Virologic Suppression in Botswana

**DOI:** 10.1371/journal.pone.0020010

**Published:** 2011-06-15

**Authors:** Katherine T. Steele, Andrew P. Steenhoff, Craig W. Newcomb, Tumelo Rantleru, Rudo Nthobatsang, Gloria Lesetedi, Scarlett L. Bellamy, Jean B. Nachega, Robert Gross, Gregory P. Bisson

**Affiliations:** 1 University of Pennsylvania School of Medicine, Philadelphia, Pennsylvania, United States of America; 2 Botswana-University of Pennsylvania Partnership, Gaborone, Botswana; 3 Division of Infectious Diseases, The Children's Hospital of Philadelphia, Philadelphia, Pennsylvania, United States of America; 4 Center for AIDS Research, University of Pennsylvania, Philadelphia, Pennsylvania, United States of America; 5 Center for Clinical Epidemiology and Biostatistics, University of Pennsylvania School of Medicine, Philadelphia, Pennsylvania, United States of America; 6 Departments of International Health and Epidemiology, Johns Hopkins Bloomberg School of Public Health, Baltimore, Maryland, United States of America; 7 Division of Infectious Diseases, University of Pennsylvania School of Medicine, Philadelphia, Pennsylvania, United States of America; McGill University, Canada

## Abstract

**Background:**

Adverse outcomes occurring early after antiretroviral therapy (ART) initiation are common in sub-Saharan Africa, despite reports of high levels of ART adherence in this setting. We sought to determine the relationship between very early ART adherence and early adverse outcomes in HIV-infected adults in Botswana.

**Methods:**

This prospective cohort study of 402 ART-naïve, HIV-infected adults initiating ART at a public HIV clinic in Gaborone, Botswana evaluated the relationship between suboptimal early ART adherence and HIV treatment outcomes in the initial months after ART initiation. Early adherence during the interval between initial ART dispensation and first ART refill was calculated using pill counts. In the primary analysis patients not returning to refill and those with adherence <0.95 were considered to have suboptimal early adherence. The primary outcome was death or loss to follow-up during the first 6 months of ART; a secondary composite outcome included the primary outcome plus incident opportunistic illness (OIs) and virologic failure. We also calculated the percent of early adverse outcomes theoretically attributable to suboptimal early adherence using the population attributable risk percent (PAR%).

**Results:**

Suboptimal early adherence was independently associated with loss to follow-up and death (adjusted OR 2.3, 95% CI 1.1–4.8) and with the secondary composite outcome including incident OIs and virologic failure (adjusted OR 2.6, 95% CI 1.4–4.7). However, of those with early adverse outcomes, less than one-third had suboptimal adherence and approximately two-thirds achieved virologic suppression. The PAR% relating suboptimal early adherence and primary and secondary outcomes were 14.7% and 17.7%, respectively.

**Conclusions:**

Suboptimal early adherence was associated with poor outcomes, but most early adverse outcomes occurred in patients with optimal early adherence. Clinical care and research efforts should focus on understanding early adverse outcomes that occur despite optimal adherence.

## Introduction

Death in the initial months after initiating combination antiretroviral therapy (ART), often termed “early mortality,” accounts for the majority of all first-year deaths in adult HIV treatment programs in resource-limited settings [1]–[3]. However, published data indicate that ART adherence is high in the very settings most affected by high rates of early mortality [4]. This suggests that classic mechanisms of HIV treatment failure, whereby suboptimal ART adherence leads to AIDS progression and death, may not predominate as a cause of early events.

More broadly, adherence as well as virologic and immunologic responses to ART among patients who suffer early mortality after ART initiation remains largely uncharacterized, in part because many events occur before these measures can be obtained. Nonetheless, understanding response to ART in these individuals is important for the design of interventions aimed at reducing early mortality. For example, patients may have difficulty tolerating medications early during therapy, leading to failure to respond virologically and immunologically and subsequent disease progression [5]. Alternatively, patients may experience poor outcomes despite high adherence levels, perhaps due to suboptimal immunologic responses despite virologic suppression [6], rapid immune response and inflammation [7], [8], drug toxicity or other factors [9]. Landmark studies documenting the importance of initial ART response to subsequent survival have usually measured initial response several months after ART initiation, after the majority of very early deaths occur, and therefore do not inform the relationship between virological and immunological response to ART and early mortality [10], [11]. Thus, a fundamental understanding of how response to ART relates to early mortality is needed to improve programmatic outcomes overall.

Adherence is strongly related to clinical outcomes and is the first step in responding to ART [12]. However, longitudinal studies evaluating adherence-outcome relationships often by design require minimum follow-up time to be included in analyses, which excludes patients who die or are lost to follow-up early after ART initiation [13]–[18]. As a result, estimates of the strength of the relationship between initial ART adherence and risk of early outcomes, as well as the proportion of early events associated with suboptimal early adherence, have not been well characterized. If the proportion of early adverse outcomes within a population that are attributable to early suboptimal adherence is low, then patient care and research efforts should include a focus on factors besides adherence when assessing patients who clinically worsen in the early stages of treatment.

In previous studies in Botswana we have documented that the vast majority of deaths in the first year after starting ART occur in the initial months of treatment and that the median adherence level measured approximately 6 months after ART initiation is high (91%) [13], [19]. We therefore hypothesized that most early deaths were occurring despite high levels of adherence and, potentially, virologic response. To investigate this further, we evaluated the relationship between the earliest routinely available objective measure of early ART adherence and risk of early adverse outcomes after ART initiation in a sub-Saharan African setting. Goals of our analysis were to include patients who suffered events very early after ART initiation in order to obtain a range of risk estimates within which the true relationship between early adherence and early outcomes is likely to exist, to estimate the proportion of early events in the population potentially attributable to suboptimal early adherence, and to describe virologic responses among those suffering early adverse outcomes.

## Methods

### Study Setting and Participants

This prospective observational cohort study evaluated the relationship between early suboptimal adherence and early adverse outcomes among ART-naïve, HIV-infected adults aged 18 years and older initiating free ART at the Infectious Disease Care Center (IDCC) at Princess Marina Hospital (PMH) in Gaborone, Botswana. The IDCC at PMH is a large public HIV clinic where HIV-infected individuals with CD4 counts <200 cells/mm^3^ or a qualifying opportunistic illness (OI) were provided with free ART [19], [20]. Patients saw medical doctors with training in HIV care, including diagnosis and treatment of OIs. Initial ART regimens recommended as first-line therapy were fixed-dose combination zidovudine-lamivudine plus either efavirenz (for women of non-reproductive potential or men) or nevirapine, although other ART medications were available. ART was dispensed on-site. Guidelines recommended HIV-1 RNA levels (viral loads) at baseline, 3 months and then every 6 months thereafter. All care was provided free of charge.

### Early Adherence and Outcome Definitions

Early adherence was determined using pill counts performed by study staff at the time of initiation and first ART refill, given data indicating that pharmacy adherence measures such as pill counts consistently predict outcomes and are more accurate than self-report [12]. Specifically, the number of days of ART dispensed to the patient at the time of starting ART was calculated using the prescribed daily dose and the number of pills of a single index ART medication in the regimen (e.g., efavirenz for those on an efavirenz-containing ART regimen). The number of days of the index medication that the patient returned with at the time of the first ART refill was then determined by pill count and subtracted from the number of days of ART initially dispensed to arrive at the estimated number of days of ART taken between initiation and first ART refill. The number of days of ART taken during this interval was then divided by the number of days between the date of ART initiation and the actual date of the first ART refill to arrive at the estimated early ART adherence. Patients were considered to have suboptimal early adherence (present or absent) if their early adherence value was <0.95 based on previous research relating refill adherence to virologic failure in this setting [13].

Loss to follow-up or death after ART initiation were considered together as the primary composite outcome given a strong documented relationship between loss to follow-up and death in this specific setting as well as several others [19], [21]. Secondary outcomes evaluated included death alone (ie, not counting loss to follow-up as an outcome) and a composite outcome including virologic failure or incident OIs in addition to the primary outcome. Loss to follow-up was defined as failure to locate the patient before completion of the study after 3 phone calls to the patient or their contacts on separate days, one home visit and absence of patient interactions with other area clinics. Virologic failure was defined as failure to achieve a viral load <1,000 copies/mL either a) after at least 3 months of ART or b) for those with more than 1 viral load measured at least 3 months after ART, at the time of the last viral load measurement. Incident OIs were defined as any World Health Organization (WHO) clinical stage 3 or 4 illness newly diagnosed after the baseline visit [22].

### Data Collection

Study staff approached patients during clinic visits. Patients providing written informed consent in English or Setswana were assessed prior to ART initiation and then monthly thereafter until approximately 6 months after ART initiation. Practically, patient follow-up was considered complete when individuals had completed the baseline visit plus 5 monthly follow-up visits scheduled after ART initiation. Data on viral loads, CD4+ T cell (CD4 cell) counts and clinical diagnoses were abstracted from paper or electronic medical records using study-specific forms. ART medication names, doses, number of pills dispensed and returned and dates of dispensation were obtained from the IDCC pharmacy. The date of loss to follow-up was the date of the patient's last contact with the clinic, laboratory or pharmacy. Greater than 90% of patients provided a cell phone number and home address where they could be reached, and patients were actively traced via phone if they missed a clinic visit. If patients were unable to be reached by phone on three separate days, the study team performed a home visit in an effort to locate the patient. Deaths and dates of death were confirmed by the patients' medical records or by patient contact report. Records of patients with incident OIs were reviewed by study staff blinded to patient adherence status to ascertain if the OI was under evaluation prior to ART initiation. If this blinded review suggested that the OI was present at baseline but undiagnosed (e.g., sputum smear was sent at a baseline visit for cough but not returned positive until a later visit), it was categorized as prevalent. Diagnoses of TB and other OIs were not always confirmed microbiologically. Data were entered into a Microsoft Access database using double data entry.

### Data Analysis

Patients were included in the analyses if they had non-missing baseline initial ART regimen data of start date, pills dispensed, drug names and doses. The vast majority of deaths in the first year after ART initiation occur in the first several months after treatment is begun, and mortality rates among those who survive this initial early treatment period decrease substantially thereafter [1]. Thus, early mortality can be thought of as a dichotomous outcome that either occurs or does not, and time to early mortality, which could be evaluated using survival analyses, is less relevant. Thus, adherence and baseline characteristics were related to primary (death or loss to follow-up) and secondary adverse outcomes (death, loss to follow-up, incident OI, or virologic failure) using relative risks (RR) with 95% confidence intervals (CI). For patients with multiple secondary adverse outcomes, the initial adverse outcome was used for the analysis. A secondary analysis also evaluated the results when limiting the primary outcome to death (ie, not counting loss to follow-up as an event). Potential confounders included any variable present at baseline and associated with either adherence or outcome at a p-value of 0.20 or less. These factors were to be included in a final logistic regression model if their inclusion changed the unadjusted odds ratio (OR) for adherence and outcomes by 15% or more. We chose to perform logistic regression rather than methods for survival analysis because our primary aim was to relate occurrence of early adverse outcomes to early adherence during the initial period of ART, and was not to compare time-to-event by adherence.

A challenge inherent in determining the relationship between early adherence and early outcomes is that some patients initiate ART and never return, in which case their adherence is unknown. Requiring patients to return for at least one ART refill in order to be included in the study would eliminate such patients from the analysis but could introduce potentially severe selection bias due to the relationship between loss to follow-up and death [21]. We therefore chose to include these individuals, and in the primary analysis the adherence of patients who initiated but never returned for ART was imputed as <0.95 (suboptimal), which is a “missing = failure” approach taken by others [23], [24]. To evaluate the effects of this assumption on the results we then performed a sensitivity analysis repeating the primary analysis after recategorizing these patients' adherence as ≥0.95 (optimally adherent). Because all patients who initiated and never returned were counted as outcomes, the primary analysis counting these patients as suboptimally adherent was considered representative of the strongest likely relationship between early adherence and early adverse outcomes in the cohort.

Most studies relating adherence to outcomes have expressed this relationship in terms of odds ratios, relative risks or relative hazards [12]. These measures of association express the etiologic relationship between exposure and outcome but do not convey the overall public health importance of a risk factor to occurrence of an outcome at the population level. To estimate the percentage of early adverse outcomes theoretically attributable to suboptimal early adherence, we calculated a population attributable risk percentage (PAR%) using the equation: PAR% = {[P_exp_(RR−1)]/[P_exp_(RR−1)+1]} * 100 where P_exp_ = prevalence of suboptimal adherence in the population, and RR = unadjusted relative risk of early adverse outcomes among patients with suboptimal adherence [25]. In this case, PAR% represents the percentage of early adverse outcomes in the entire cohort that theoretically would not have occurred if suboptimal early adherence was completely eliminated [26]. We also described the proportion of patients with specific initial adverse outcomes (e.g., death, incident OI) who achieved a viral load ≤400 copies/mL during follow-up, as well as the timing of their virologic response in relation to their initial outcomes. Finally, data on illnesses considered contributing to death among those who died were collected from patient medical records or patient contacts.

Analyses were done using SAS 9.1 (SAS Institute Inc.), Cary, North Carolina, USA. The research ethics boards of the Botswana Ministry of Health, the PMH and the University of Pennsylvania approved this research protocol.

## Results

### Characteristics of the cohort

Between May 31, 2005 and November 19, 2007, 474 patients were enrolled. Of these, 40 (8%) were later found to be ineligible because they were not ART naïve, 28 (6%) had missing initial ART regimen data and 2 (<1%) withdrew, leaving 402 (85%) for analysis. The median date of enrollment was December 31, 2006 and the median follow-up time was 256 days (interquartile range (IQR) 180 to 344 days), reflecting the observed variability in actual time to completing 5 follow-up visits in the study. Baseline characteristics of the cohort are shown in Table 1. The median baseline CD4 count was 132 cells/mL^3^ (IQR 73–180). Forty of 45 patients (89%) being treated for active TB at baseline, and 288 of 321 patients (90%) who were not being treated for active TB and who had a baseline CD4 count <200 cells/mm^3^ were on trimethoprim-sulfamethoxazole at the time of ART initiation. Furthermore, 94 of 275 patients (34%) who were not being treated for active TB and who did not have a prior history of isoniazid preventive therapy (n = 84) were given isoniazid at baseline. Among 367 patients (91%) with non-missing baseline hemoglobin values, the median hemoglobin level was 11.5 gm/dL (IQR 10.0–13.1).

**Table 1:** Baseline characteristics, initial ART days dispensed, and initial adverse outcomes among patients in the prospective study (n = 402).

Female sex (n, %)	250 (62%)
Age (mean, range in years)	37 (range, 19 to 69)
Median CD4 count (cells/mm^3^, IQR)	132 (IQR 73–180)
Median hemoglobin (gm/dL, IQR)	11.5 (IQR 10.0–13.1)
Any baseline WHO stage 3 or 4 OI (n, %)	89 (22%)
On anti-tubercular therapy (n, %)	52 (13%)
Baseline use of trimethoprim and sulfamethoxazole (n, %) among 366 eligible patients^a^	328 (90%)
Baseline use of isoniazid preventive therapy (n, %) among among 275 eligible patients^b^	94 (34%)
Initial ART regimen	
AZT/3TC/EFV (n, %)	212 (53%)
AZT/3TC/NVP (n, %)	171 (43%)
d4T/3TC/EFV (n, %)	2 (1%)
d4T/3TC/NVP (n, %)	11 (3%)
Other (n, %)	6 (1%)
Initial ART days dispensed (median days, IQR)	20 (IQR 20–35)
Time to first ART refill (median days, IQR)	19 (IQR 14–30)
Suboptimal early adherence (n, %)	75 (19%)
Initial adverse outcomes - primary definition (n, %)	
Lost to follow-up	9 (2%)
Death	28 (7%)
Initial adverse outcomes - expanded definition (n, %)	
Virologic failure	8 (2%)
Incident OI	34 (8%)
Lost to follow-up	8 (2%)
Death	14 (3%)

OI = opportunistic illness; ART = combination antiretroviral therapy; IQR = inter-quartile range.

Percents may not add up to 100% due to rounding.

a Patients considered eligible for trimethoprim-sulfamethoxazole included patients being treated for active TB at baseline and patients not being treated for active TB who had a baseline CD4 count <200 cells/mm^3^.

b Patients considered eligible for isoniazid preventive therapy included those who did not have known active TB and who did not have a prior history of isoniazid preventive therapy.

Overall, 37 of 402 patients died (n = 28, 7%) or were lost to follow-up (n = 9, 2%), and the median time-to-event among patients who died was approximately 3 months (104 days, IQR 52 to 196 days). Table 2 shows the myriad diagnoses made in the patients who died. Of the 37 deaths and losses to follow-up, 9 (24%) never returned for an ART refill after ART initiation (6 died and 3 were lost to follow-up). When expanding the outcome to the composite definition including virologic failure and incident OIs, 64 of 402 patients (16%) had an initial early adverse event after ART initiation. Over half of these initial outcomes were incident OIs (Table 1), and TB was the most common incident OI (n = 9). The median number of initial ART regimen days dispensed was approximately 3 weeks (Table 1), and 75 of 402 (19%) patients had suboptimal early adherence (median adherence = 1, IQR 0.96 to 1). The median adherence of those with suboptimal adherence was 0.86 (IQR 0.76–0.94).

**Table 2:** Primary Cause of Death Among Patients who Died During the Study Period (n = 28).

Cause of Death	n (%)
Pulmonary Tuberculosis (PTB)	3 (11)
Hepatitis / Hepatic Encephalopathy	2 (7)
Meningitis (Unknown etiology)	2^a^ (7)
Cryptococcal Meningitis	1^b^ (4)
TB Adenitis	1 (4)
Pneumocystis Pneumonia (PCP)	1 (4)
Pneumonia	1 (4)
Pancytopenia / Severe Wasting Syndrome	1 (4)
AZT-induced Anemia	1^a^ (4)
Anemia	1^b^ (4)
Pericardial Effusion	1^b^ (4)
Encephalitis	1 (4)
Stroke / Multiple intracranial Abscesses	1 (4)
AIDS-related global dementing process vs. Multiple brain lesion pathology	1 (4)
Sepsis	1 (4)
Anasarca secondary to hypoalbuminemia	1 (4)
Unknown cause	8 (29)

a Patients had a diagnosis of Kaposi's sarcoma which may have contributed to death.

b Patients had a diagnosis of gastritis which may have contributed to death.

Table 3 shows relationships between baseline characteristics, early adherence and early outcomes. As expected, patients with the lowest CD4 counts were at increased risk of loss to follow-up and death [27], although patients with OIs that were diagnosed at baseline were not (Table 3). Adverse early outcomes were significantly more common among those who had evidence of suboptimal early adherence compared to those who did not [16.0% vs 7.6%, RR 2.09 (95% CI 1.10–3.97) and 28.0% vs 13.1%, RR 2.13 (95% CI 1.35–3.36) for primary and secondary outcomes, respectively] (Table 3). This relationship was unchanged when only deaths and not losses to follow-up were counted as outcomes [RR 2.11, 95% CI 1.00–4.47)]. No confounders of these relationships were identified after evaluating all of the variables listed in Table 1 (including baseline OIs and baseline CD4 count evaluated as either a continuous or categorical variable) and therefore adjusted analyses were not required. In sensitivity analyses, reclassifying patients who initiated but never refilled ART as adherent decreased the prevalence of suboptimal adherence from 75 of 402 (19%) to 66 of 402 (16%) and changed the adherence-outcome relationship so that it was no longer statistically significant [RR 0.45 (0.14–1.42) and 1.17 (0.66–2.08) for primary and secondary outcomes, respectively].

**Table 3:** Unadjusted relative risks of adverse early outcomes according to patient characteristics.

Characteristic	Group	Primary outcome	RR (95% CI)	Secondary outcome	RR (95% CI)
Sex	Female (n = 250)Male (n = 152)	18 (7.2%)19 (12.5%)	Reference1.74 (0.94–3.20)	34 (13.6%)30 (19.7%)	Reference1.45 (0.93–2.27)
Age	≤35 (n = 202)>35 (n = 200)	17 (8.4%)20 (10.0%)	Reference1.19 (0.64–2.20)	28 (13.9%)36 (18.0%)	Reference1.30 (0.83–2.04)
Baseline CD4 count	≤50 (n = 66)51–150 (n = 166)>150 (n = 167)	10 (15.2%)14 (8.4%)11 (6.6%)	2.30 (1.03–5.16)1.28 (0.60–2.74)Reference	13 (19.7%)25 (15.1%)24 (14.4%)	1.37 (0.74–2.53)1.05 (0.62–1.76)Reference
Baseline OI	Absent (n = 313)Present (n = 89)	28 (8.9%)9 (10.1%)	Reference1.13 (0.55–2.31)	45 (14.4%)19 (21.3%)	Reference1.48 (0.92–2.40)
Baseline active TB ATT	Absent (n = 357)Present (n = 45)	36 (10.1%)1 (2.2%)	Reference0.22 (0.03–1.57)	60 (16.8%)4 (8.9%)	Reference0.53 (0.20–1.39)
Initiated EFV-based ART	No (n = 188)Yes (n = 214)	15 (8.0%)22 (9.2%)	Reference1.29 (0.69–2.41)	26 (13.8%)38 (17.8%)	Reference1.28 (0.81–2.03)
Early adherence	Optimal (n = 327)Suboptimal (n = 75)	25 (7.6%)12 (16.0%)	Reference2.09 (1.10–3.97)	43 (13.1%)21 (28.0%)	Reference2.13 (1.35–3.36)

OI = opportunistic illness; ATT = anti-tubercular therapy for active tuberculosis; TMP/SMX = trimethoprim-sulfamethoxazole; EFV = efavirenz; RR = relative risk; Primary outcome = death or loss to follow-up; Secondary outcome = incident OI, virologic failure, loss to follow-up or death.

Using death or loss to follow-up as the outcome, the unadjusted RR for suboptimal early adherence and this outcome of 2.09, and the prevalence of suboptimal early adherence of 0.19, the PAR% for early death or loss to follow-up from suboptimal early adherence was 14.7%. For the composite outcome including OIs and virologic failure, the PAR% was 17.7%.

Approximately two-thirds of patients who experienced early outcomes had early adherence ≥95% [25 of 37 (68%) and 43 of 64 (67%) for primary and secondary outcomes, respectively], and the high levels of adherence, even in those with early outcomes, were reflected in high rates of virologic response. Overall, 355 of 402 (88%) achieved at least one viral load ≤400 copies/mL. Among the 56 patients who had initial adverse clinical outcomes that were not virologic failure, 24 (43%) achieved one or more viral loads ≤400 copies/mL prior to or on the day of their initial adverse outcome, 10 (18%) achieved this degree of virologic response after the initial outcome and 22 (39%) did not have a documented viral load at or below this level. Of these 22, only 1 patient had documented virologic failure, while the remaining 21 did not have follow-up viral load measurements. According to initial event type, 68% (23 of 34) of those whose initial event was an incident OI, 57% (8 of 14) of those whose initial and only recorded event was death, and 61% (34 of 56) of those with any initial non-virological adverse outcome had one or more documented viral loads ≤400 copies/mL. The median time between the closest viral load ≤400 copies/mL and the initial adverse outcome among those who had non-virologic initial adverse outcomes and measured viral loads was 3 days (IQR 68 days before to 39 days after).

## Discussion

This study, based on prospectively collected data from routine clinical practice in Botswana, provides evidence that suboptimal early ART adherence as measured by pharmacy refill data and pill counts increases the risk of early adverse outcomes. In the primary analysis, patients who had suboptimal early adherence during approximately the first 21 days of ART had twice the risk of early adverse outcomes as compared to patients whose adherence was ≥95%. However, the proportion of early adverse outcomes theoretically attributable to suboptimal early adherence in this population, estimated as the PAR%, was relatively low (<20%), and most early outcomes observed occurred in patients with high levels of adherence and virologic suppression around the time of their event. These results do not challenge the fact that adherence is critically important in HIV care. However, they do indicate that in many individuals living in similar settings with similarly advanced HIV disease, even high levels of initial ART adherence may not be enough to prevent emergent OIs, AIDS progression, and death. The findings provide further data supporting the revised WHO guidelines stressing the need to start ART at earlier stages of HIV [28].

Our finding of increased risk of events in patients with suboptimal adherence are consistent with a similar study from Kenya which found an increased risk of death and loss to follow-up in patients with lower adherence measured at 2 months post-ART initiation [23] and more broadly with studies relating adherence to survival over longer periods of time [17], [29]. Considered together with studies indicating that both adherence and early mortality are higher in sub-Saharan Africa compared to industrialized countries [4], [30], these data suggest that AIDS progression and death in the setting of high levels of ART adherence and virologic suppression is likely common in the region. One reason for this is likely related to difficulty diagnosing OIs that are present but untreated at baseline. Indeed, the finding that the majority of non-death early adverse outcomes were incident WHO grade 3 and 4 OIs indicates that improving diagnosis and treatment of OIs around the time of ART initiation has the potential to meaningfully reduce early adverse outcomes, as has been suggested by others [31], [32]. The fact that we did not find an increased risk of early adverse outcomes among those who had OIs that were diagnosed at ART initiation is similar to results from studies finding no increased risk of early mortality in patients with known pulmonary TB at ART initiation [31], [33] and further supports efforts to detect and treat these conditions at the time of starting ART. The high proportion of initial outcomes that were OIs also warrants further consideration of “unmasking” immune reconstitution inflammatory syndrome (IRIS) [34], [35] as a potentially important mechanism of early clinical deterioration. Although recent data from sub-Saharan Africa indicate that fatal IRIS is rare [8], [36], patients may die before excessive inflammation can be proven, particularly when inflammation occurs very early after ART initiation and when studies are retrospective. Therefore further evaluation of the role of very early inflammation in early morbidity and mortality is warranted.

We did not prospectively grade drug toxicity, but many ART drugs including zidovudine and nevirapine, which were initiated by most and nearly half of the patients in this study, respectively, have potentially fatal toxicities that could cause early mortality despite and perhaps even due to high levels of adherence [37], [38]. Severe drug toxicity in treatment centers in Africa is well-described [39], and both zidovudine-associated anemia [9] and lactic acidosis [40] may be more common in treated HIV-infected Africans. More broadly, clarifying the mechanisms by which adverse outcomes occur in the setting of high adherence and virologic suppression, of which drug toxicity is one, should be pursued.

Strengths of this study include a novel focus on early adherence data using prospective prescription information, careful ascertainment of data on incident OIs, active patient tracing to determine outcomes, the sub-Saharan African setting and the availability of routinely-collected viral load results, which enhanced our evaluation. Inclusion of patients who initiated but never returned for an ART refill in the primary analysis is another strength of the study. Given that risk of early mortality after ART initiation in resource-limited settings is highest very early after starting ART [30], excluding individuals who are lost to follow-up or die early after ART initiation excludes precisely those to whom the study question is most relevant. In contrast, including these individuals and using sensitivity analyses to provide an estimate of the strongest and weakest likely relationships between early adherence and early adverse outcomes provides a more comprehensive evaluation of the association. While it is more reasonable to assume that patients who initiated but never returned to refill ART were not optimally adherent, it is also possible that some adhere up to their final days and some discontinue very early, in which case the true relative risk for early adherence and early adverse outcomes is somewhat weaker, in an epidemiologic sense, than was determined in the primary analysis.

Our study also has several limitations. For example, the generalizability of our findings of relative risk and the low PAR% could be limited if the patients agreeing to participate in this study were more adherent compared to those being treated in other settings. The overall rate of documented virologic response in the cohort (88%) was slightly higher than pooled estimates from sub-Saharan Africa (76%), suggesting that the study sample demonstrated adherence that was relatively high but certainly not outside of observed ranges [41]. Furthermore, we relied on information obtained via routine care, and more clinical details could have better informed the design of interventions aimed at reducing early outcomes. Another limitation is that some patients were lost to follow-up, and therefore we cannot be certain that they experienced an adverse outcome after ART initiation. However, most patients lost to follow-up were lost very soon after ART initiation and shorter time to loss to follow-up is associated with increased risk of mortality among those who are lost from care [21]. Patients lost to follow-up from this public treatment program have previously been shown to have a high rate of mortality, which further suggests that those classified as lost in this study had poor outcomes [19]. Furthermore, major bias due to loss to follow-up did not occur because exclusion of patients who were lost did not change the study findings. Another limitation relates to the fact the cause of death was not known for 8 (29%) of the patients who died, leaving open the possibility that these individuals died from processes unrelated to their HIV infection or their adherence to antiretroviral therapy. The low median baseline CD4 count of the cohort and the fact that the majority of patients for whom the cause of death was known died from OIs, most commonly TB (n = 4, 14%), suggests that most deaths were HIV-related. Finally, our analysis of the relative risk of early outcomes among those with suboptimal early adherence inherently relates early outcomes occurring in the presence of essentially any suboptimal adherence to the suboptimal adherence itself. However, in cases where only a few doses were missed suboptimal adherence is unlikely to be the sole cause of the outcome. Viral loads decline logarithmically during a time period equivalent to that which these patients most commonly were supplied ART (i.e., 20 days) [42], and therefore the proportion of early outcomes that could be prevented by a sole focus on improving early adherence is likely even smaller than the PAR% values would suggest.

In conclusion we found that suboptimal early adherence, while associated with adverse outcomes, is a relatively uncommon finding in patients experiencing early adverse outcomes. Given the high contribution of early outcomes to morbidity and mortality in ART treatment efforts in resource-limited settings, further research to understand, identify, treat and prevent these outcomes is needed.
